# The use of cannabis for Hyperemesis Gravidarum (HG)

**DOI:** 10.1186/s42238-020-0017-6

**Published:** 2020-01-30

**Authors:** Gideon Koren, Rana Cohen

**Affiliations:** 1 Adelson Faculty of Medicine, Ariel University, 40700 Ariel, Israel; 2 Motherisk Israel Program, Shamir Hospital, Be’er Ya’akov, Israel

## Abstract

**Background:**

Hyperemesis Gravidarum (HG) responds only partially to standard antiemetic medications. Cannabis has been known to possess antiemetic effects and there are several medicinal cannabinoids used as anti -emetics for cancer chemotherapy. Its favorable use for HG has been described in social media, but not in the medical literature.

**Methods:**

We evaluated 4 women with HG counseled by the Motherisk Program, before and following the use of cannabis. Using the validated Pregnancy Unique Quantification of Emesis (PUQE) scoring system and employing the Student’s paired t test, we compared changes in symptoms following initiation of cannabis.

**Results:**

There was a highly significant improvement in symptoms: PUQE score improved from 14.5+/− 1 to 7.5+/− 0.58(*p* = 0.0004). Cannabis use was associated with a significant increase in the PUQE Quality of Life scale, from 2+/− 0.82 to 7+/− 0.82 (*p* = 0.0012).

**Conclusions:**

The results suggest that cannabis may be effective for HG, and should be studied in appropriately powered, controlled studies, fully considering potential fetal risks.

## Background

Nausea and Vomiting of Pregnancy (NVP) affect up to 80% of pregnant women and is characterized by varying severity of symptoms that in most women subside by the end of the first trimester of pregnancy. At the severe end of NVP, Hyperemesis Gravidarum (HG) affects between 1 and 2% of pregnant women and is characterized by severe and protracted nausea and multiple vomiting, weight loss, dehydration and electrolyte imbalance, responding only partially to standard antiemetic medications (Dean et al., 2018). Most women experiencing HG need hospitalization for rehydration and nutritional support. Cannabis has been known to possess antiemetic effects and there are several medicinal cannabinoids used as anti -emetics for cancer chemotherapy (Mersiades et al., 2018). A narrative review of PubMed, EMBASE, Cochrane and social media has revealed large numbers of American women claiming favorable effects of cannabis on their HG symptoms (Facebook, 2019). However, these uncontrolled reports are difficult to evaluate due to the unstructured methods of data collection and reporting. In parallel, several epidemiological studies have reported on cannabis use in pregnancy. In Hawai, 2.6% of women reported using marijuana during pregnancy between 2009 and 2011. The 21.2% of women who reported severe nausea during pregnancy were significantly more likely to report marijuana use during gestation (Robertson et al., 2014). To the best of our knowledge, to date there has not been direct clinical research documentation of cannabis effects in HG. We describe four cases of cannabis use among women who called and were followed up by a medical a service focusing on providing information and counseling for pregnant women suffering from HG .

## Methods

This retrospective study was approved by Shamir Hospital Research ethics board in Israel. All women consented to including their cases in the article, based on anonymous presentation. The study was conducted by the Motherisk Program, an academic counseling and follow- up service for women experiencing NVP and HG. Counseling and follow up telephone interviews are conducted by a trained counselor who has expertise in pharmacy and business administration. The counselor is backed by two pediatric pharmacologists/ reproductive toxicologists and a large team of drug information specialists. The counseling sessions include advice on nutrition, fluid intake, medicinal and non –medicinal therapeutic interventions. In this study, we analyzed cases followed up by us after using cannabis, which was initiated by the women, with support of their physicians (who did not prescribe to cannabis). For each woman we recorded the symptoms of nausea (length and strength) vomiting (numbers) and retching (number) and scored them based on the validated PUQE score (Koren et al. 2002) (Table 1). We scored the PUQE score and its Quality of Life scale reported before cannabis was started, and subsequently after at least 3 days of use. The PUQE score ranges from 3 (no symptoms) to 15 (maximal symptoms). The Quality of life scale is based on a visual analogue scale, ranging from 0(the worst possible) to 10 (best) PUQE Score (from Koren et al., 2002).

**Table 1:**
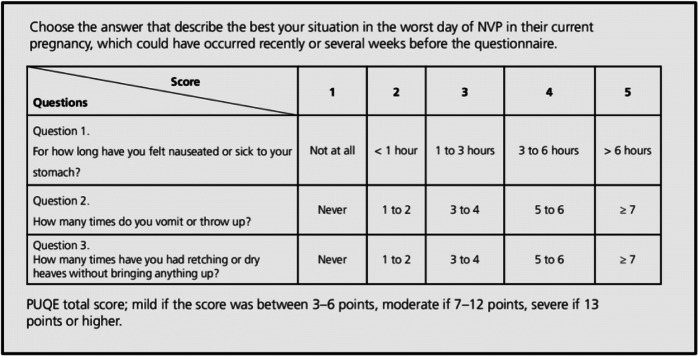
PUQE Score (from Koren et al., 2002)

The PUQE scores before and after starting cannabis were compared by the Student’s t test for paired data. The Kolmogorov –Smirnov test of Normality was used to verify whether the data were normally distributed, with calculation of Skewness and Kurtosis.

## Results

### Cases

1. A 32 year old physician in her third pregnancy. Her first pregnancy was characterized by HG between weeks 6–20, with no response to Diclectin (doxylamine and pyridoxine) and partial response to intravenous ondansetron. A healthy 3.4 Kg baby boy was born at 40 weeks. Her second pregnancy was also complicated by HG between weeks 6–16, again with no response to Diclectin and partial response to ondansetron. A healthy 3.14 Kg aby girl was born at 40 weeks.

In her third pregnancy HG started at 6 weeks, responding partially to Diclectin but with a marked sedative effect. Her PUQE score before starting cannabis was at 13. Starting at 11 weeks of gestation she tried 3 different types of cannabis containing 18–23% THC and 0.8–1% CBD by smoking through a pipe. The nausea and vomiting completely resolved after 2–3 puffs and the effect was sustained for 3 h. After several weeks the response weakened, covering only 2 h. The PUQE score following cannabis was at 7. Her Quality of life score was 3 prior to cannabis and was raised to 7. Overall, she was using 1–2 g cannabis per day. Before starting the cannabis she had lost weight, which reversed with cannabis and overall she gained 7 Kg over her pregnancy. She continued with cannabis till birth due to continued symptoms. The cannabis effectively controlled vomiting but she continued to be nauseated. She gave birth to a healthy 3.4Kg 39 wk. boy at Apgar 9–10 who had a short bout of transient tachypnea of the newborn. Long term follow up by the Denver scale suggested normal development and achievement of milestones.

2) A 32 year old woman who experienced HG between weeks 6–14 of her first pregnancy. She was treated with metoclopramide but developed an allergic reaction.

In her 2nd pregnancy HG started at 4 weeks with up to 70 bouts of vomiting a day necessitating repeated hospitalizations and use of a nasogastric tube and parenteral nutrition. She was continuously hospitalized with her vomiting responding to Diclectin and ondansetron but with continued severe nausea, which prevented her from eating***.*** Her PUQE score was at 15. While continuing Diclectin and ondansetron, she started at 14 weeks of gestation smoking cannabis, trying 3 different types containing 18–20% THC and 0.1% CBD. Two-three puffs resulted in total relief of nausea and vomiting, ability to eat and keeping the food down. Each round of puffs sustained its effect for 3–4 h, and for the remaining of her pregnancy she was not hospitalized despite continuation of her symptoms. Her PUQE score decreased to 7. Her Quality of Life score increased from 2 to 7. She experienced a mild sedative effect with the different types of cannabis, and hypotension with the Sativa. She asked to be delivered at 36 weeks due to her symptoms of HG, and a healthy baby boy of 2Kg was born, who, at 1 year of age appears to meet his developmental milestones.

3) A 33 year old woman experienced mild NVP in her first pregnancy responding to metoclopramide, giving birth to a healthy girl.

In her second pregnancy she experienced HG starting at 10 weeks, unresponsive to Diclectin, ondansetron or ginger. She experienced up to 20–25 bouts of vomiting a day, extreme weakness, falls, and needed 3 hospitalizations receiving ondansetron intravenously, which controlled the vomiting but with continuous severe nausea, precluding eating. She was maintained on home-care infusions plus ondansetron.. Her PUQE score was at 15.

At 12 weeks gestation she started smoking different cannabis preparations containing 20% THC and 0.1% CBD. She also tried to use THC in sublingual drops, but with no effect.

With the combination of IV ondansetron and smoking cannabis 2–3 puffs every 2 h for a total of 1 g/d her vomiting decreased from 6 to 15 a day to only 6 a day. Her PUQE score was at 8. Her Quality of Life scale increased from 2 to 8. Her overall status improved dramatically, with significantly less depression, improved appetite and substantially less nausea. Prior to cannabis she had lost 3 Kg of body weight, but with cannabis she resumed weight gain. She continued with cannabis till one week prior to birth. A full term baby boy was born at 2.6 Kg with normal developmental trajectory.

4) A first pregnancy of a woman suffering from colitis and fibromyalgia who had been treated with medical cannabis that was stopped before pregnancy at 2 g/d as 2–3 puffs every 2–3 h.

She experienced HG starting at 7 weeks of gestation***.*** Her PUQE score was 15. The use of cannabis was associated with major improvement in nausea and vomiting. Her PUQE score improved to 8. For religious reasons she did not used cannabis on Saturdays, resulting in nausea and lack of appetite. She was also treated with 5 amino salycilic acid and citalopram for her colitis and fibromyalgia. Her Quality of Life scale improved from 1 to 6.

A healthy 2.6 Kg baby girl was born at term. At 4 years of age she is healthy and appears to be developing well.

### Statistical analysis

The PUQE and Quality of Life values in our patients followed normal distribution evidenced by the Kolmogorov-Smirnov test and calculation of skewness and kurtosis. The data did not differ significantly from that which is normally distributed, with D value of 0.41, skewness of 0 and Kurtosis of − 3. Following use of cannabis, the mean PUQE score decreased from 14.5+/− 1 (mean +/− standard deviation) to 7.5+/− 0.58 (*p* = 0.0004). Cannabis use was associated with a significant increase in Quality of Life scale, from 2+/− 0.82 to 7+/− 0.82 (*p* = 0.0012). For both comparisons variances were very close, meeting parametric assumptions.

In all 4 cases, the women experienced weight loss prior to using cannabis, which was reversed following cannabis. In 4 cases there was improved appetite. One woman reported hypotension and sedation while on cannabis.

## Discussion

In these 4 severe cases of HG, cannabis preparations containing 20%THC and traces of CBD appeared to have a dramatic effect on the course and severity of the condition. We included all 4 cases collected by us, hence there was no bias in applying statistical analysis, and despite the small numbers, the differences following cannabis were highly significant. The concentrations of the cannabis compounds were measured as part of the quality assurance programs of the official suppliers to the Ministry of Health. The mean PUQE score improved from 14.5 which is in the upper range of severity, to 7.5, corresponding to the middle range of moderate cases. To put these changes into perspective, this is a very large effect size of 7 using Cohen’ d. In studies on NVP, the typical effect of anti emetics such as diclectine has been of medium effect size. Although the cannabis was produced in several different laboratories, the ranges of concentrations of active THC and CBD were similar. Small doses of 2–3 puffs appeared to deliver optimal relief. In British Columbia, of 79 pregnant respondents, 51 (65%) reported using cannabis during their pregnancies. While 59 (77%) of the respondents who had been pregnant had experienced nausea and/or vomiting of pregnancy, 40 (68%) had used cannabis to treat the condition, and of these respondents, 37 (over 92%) rated cannabis as ‘extremely effective’ or ‘effective’ (Westfal et al., 2006). A major issue is the existence of numerous different preparations of cannabis, without appropriate comparator data among them, leaving the women with the need for trial and error. Other important issues that must be adequately addressed are the fetal safety of cannabis (Sharapova et al., 2018) and balancing it with the fetal risks of undertreated HG (El Maroun et al. 2019).

In our cases, three of the four children had evidence of growth restriction which is a typical outcome of the HG- induced severe calorie and protein restriction, in parallel to missing numerous micronutrients (Koren et al., 2018). All 4 children appeared to have developed normally although no formal testing were performed on them and no control group was available. Concerns about long term child development have been a major reason for the ACOG to warn about cannabis use in pregnancy, (Committee on bostetric practice, ACOG, 2017). Previous studies were all done among recreational users who have numerous confounders for developmental delay. This may not be the case in women using cannabis for HG. In contrast, women with HG may be needing larger doses of cannabis for longer durations than are used by recreational users. To date, after large numbers of follow up studies, there is still controversy whether fetal cannabis exposure is associated with independent developmental delays. Associations between cannabis use and offspring achievements among recreational users has been affected by residual environmental and parental confounders (Sharapova et al., 2018). In a recent review, Metz and Borgelt (2018) documented that there are many legitimate concerns regarding the safety of marijuana in pregnancy, including animal studies and biological plausibility.

Presently the safety of marijuana in pregnancy has not been demonstrated and its use is advised against by many professional societies. While it is possible that risks of untreated HG are higher than previously thought (Koren et al., 2018), this issue will have to be addressed by future research and fetal cannabis exposure cannot be assumed to be safe for HG.

## Conclusions

While more research is needed before cannabis can be considered for use in HG, this report suggests that cannabis should be tested in appropriately- powered control trials for this severe and protracted maternal condition, addressing both maternal effect and potential adverse fetal effects.

## Data Availability

The datasets used and/or analyzed during the current study are available from the corresponding author on reasonable request.

## Abbreviations

CBD: Cannabidiol
HG: Hyperemesis gravidarum
IV: Intarvenous
PUQE: Pregnancy-unique quantification of emesis
THC: Tetra hydro cannabinol
